# Recent update on XBB.1.5 emerging novel mutation of COVID-19

**DOI:** 10.1097/JS9.0000000000000254

**Published:** 2023-02-15

**Authors:** Ruhul Amin, Prosanta Pal, Kuldeep Dhama, Talha B. Emran

**Affiliations:** a Faculty of Pharmaceutical Science, Assam Down Town University, Panikhaiti, Guwahati, Assam; b Department of Pharmaceutical Technology, University of North Bengal, Darjeeling, West Bengal; c Division of Pathology, ICAR-Indian Veterinary Research Institute, Bareilly, Izatnagar, Uttar Pradesh, India; d Department of Pharmacy, BGC Trust University Bangladesh, Chittagong; e Department of Pharmacy, Faculty of Allied Health Sciences, Daffodil International University, Dhaka, Bangladesh

Highlights

- XBB.1.5 variant may improve the virus’s ability to connect to cells.
- The XBB.1.5 sublineage was discovered in the United States.
- The XBB.1.5, the strain detected in eight patients in India.
- The subvariant has an uncommon mutation that might increase its infectiousness.

*Dear Editor*,

The emergence of the XBB family of variations during the last few months has piqued the interest of virologists since this family has more mutations than any other to circumvent immunity^1^. A mutation in the XBB.1.5 variant may improve the virus’s ability to connect to cells and spread to new hosts.

According to estimates from the Centers for Disease Control and Prevention, USA, the XBB.1.5 subvariant currently accounts for roughly 28% of US coronavirus disease 2019 cases, and its frequency is growing worldwide^2^. It seems to have quickly outcompeted a variety of other immunity-evading strains predicted to circulate together this winter in the Northeastern United States.

The XBB.1.5 sublineage was discovered in the United States in October 2022 and has subsequently been found in numerous additional countries, including South Africa, for the first time in a sample obtained at the end of December 2022. It was predicted that XBB.1.5 would eventually be found in South Africa, given its increasing global prevalence^3^. In the United States, XBB.1.5 has been demonstrated to have a growth advantage over older versions, suggesting that it may outcompete other variants to become the predominant circulating variant in various circumstances. This branch of the family tree has been designated as a ‘subvariant of concern’ and is currently being tracked. The increased binding of the virus to the human ACE2 receptor is assumed to be the cause of the growth advantage, which in turn boosts viral transmissibility. This alteration (F486P) is located in the spike protein^4^.

The XBB.1.5 strain has already been detected in eight patients in India, according to data compiled by the Indian SARS-CoV-2 Genomics Consortium (INSACOG). They reported a new case of this strain was discovered in Uttarakhand on 09 January 2023, adding to the three cases previously reported from Gujarat and one case reported from each of Karnataka, Telangana, Chhattisgarh, and Rajasthan^5^.

The scientific community has come to the consensus that XBB.1.5, like its progenitor XBB, is an expert at evading the immune system. Vaccines and infections, even with older Omicron strains, are less effective because of their numerous spike mutations. Bivalent vaccines have been demonstrated to boost levels of antibodies that may prevent infection with XBB in the lab and are likely used in XBB.1.5 prevention^6^,^7^.

At this time, it is unknown whether the degree of infection will vary across the various Omicron sublineages. The WHO Technical Advisory Group on Virus Evolution is now performing an ongoing risk assessment on XBB.1.5. Researchers argue that careful lineage tracking is necessary whether or not XBB.1.5 results in significant coronavirus disease 2019 waves. The subvariant has an uncommon mutation that might increase its infectiousness and provide a selective advantage for future evolution.

## Ethical approval

Not applicable.

## Sources of funding

No funding was received.

## Author contribution

R.A.: conceptualization, data curation, writing – original draft preparation, and writing – reviewing and editing. P.P. and K.D.: data curation, writing – original draft preparation, and writing – reviewing and editing. T.B.E.: writing – reviewing and editing, visualization, and supervision.

## Conflicts of interest disclosure

The authors declare that they have no conflicts of interest.

## Research registration unique identifying number (UIN)

1. Name of the registry: not applicable.
2. Unique identifying number or registration ID: not applicable.
3. Hyperlink to your specific registration (must be publicly accessible and will be checked): not applicable.

## Guarantor

Talha Bin Emran, PhD, Associate Professor, Department of Pharmacy, BGC Trust University Bangladesh, Chittagong 4381, Bangladesh. Tel: +88 030 335 6193, fax: +88 031 255 0224. https://orcid.org/0000-0003-3188-2272.

## Provenance and peer review

Not commissioned, internally peer-reviewed.

## Data availability

The data in this correspondence article is not sensitive in nature and is accessible in the public domain. The data is, therefore, available and not of a confidential nature.
